# The Theories of Immunity1A paper read before the Virginia State Veterinary Medical Association, Richmond, Va., January 2, 1896.

**Published:** 1896-03

**Authors:** E. P. Niles

**Affiliations:** Blacksburg, Va.


### THE THEORIES OF IMMUNITY.[1]

BY E. P. NILES, D.V.M.,
BLACKSBURG, VA.

No branch of the science of biology is receiving at the present time more deserved attention than immunity. It may also be said that less is known about this part of the science than of any other.

It is not my intention to offer anything new on the subject. If I even succeed in placing before you in an intelligent manner the views of our eminent bacteriologists I shall feel that my feeble effort has not been in vain. In the beginning of this paper I shall not attempt to offer a definition for the term immunity, but shall confine my remarks to a discussion of the theories of immunity and a few of the experiments brought to bear in support of the same.

Immunity may be discussed under two heads, viz., natural and acquired. It is a well-known fact that certain bacterial diseases affect a certain class of animals, while others are exempt from the action of these germs. Glanders is essentially a disease of the equine species, while the bovine species show perfect immunity to the disease. The dog and rat are decidedly immune to anthrax, while it is readily communicated to the equine, bovine, and human races by inoculation. Even age in animals of the same species seems to influence the degree of immunity. In the human family scarlet fever and whooping-cough are considered children's diseases, since they are much more prevalent in them than in adults. Diphtheria is also more frequently met with in children. At the same time, however, I have heard physicians assert that a young baby will not contract the disease. If this be true it can only be explained by the supposed germicidal action of the thymus gland, which rapidly disappears after birth, and possibly some influence of the mother's milk. In the lower animals anthrax is known to be much more fatal to the young, while in Texas cattle fever the old seem to be more susceptible.

It is said that the blood of naturally immune animals when

[1] A paper read before the Virginia State Veterinary Medical Association, Richmond, Va., January 2, 1896.

injected into susceptible animals will counteract the effect of certain disease-produing germs. For instance, a small quantity of the blood of a frog when injected into the circulation of a mouse will render that animal immune to anthrax, but has no such action in reference to other diseases. It would seem, then, that an animal or person may be immune to certain diseases and that the blood of such an individual has, to a certain extent, the property of producing immunity in other subjects.

Just what this immunitive substance is remains to be discovered. Many theories have been advanced, but they all lack positive proof.

Before discussing these theories in detail it must be remembered that in susceptible animals pathogenic germs, when introduced into the system, multiply with great rapidity, while in non-susceptible animals no such multiplication takes place.

In naturally immune animals, then, there must be some substance which prevents this development of the germ, or some condition of the system unfavorable to its growth.

In some the body temperature seems to be the unfavorable element, since certain bacteria only grow in very narrow limits of temperature. The common fowl is generally supposed to be immune to anthrax under normal conditions, but Pasteur has shown that they may be rendered susceptible by being refrigerated after inoculation, by immersing their bodies in cold water. On the other hand, Koch has pointed out that sparrows, whose normal temperature is as high as that of the fowl, contract the disease without refrigeration. From the above we are led to the conclusion that the high temperature of the fowl is not the only factor brought to bear in rendering it immune to anthrax. Gibier has shown that the anthrax bacillus will multiply in both the frog and fish if kept in water at a temperature of 35° C.; both being immune under ordinary circumstances. The bacillus of anthrax, however, grows under quite a wide range of temperature, and it is more than probable that some agent other than the body temperature of the subjects mentioned has something to do in causing this natural immunity. There is, however, certain pathogenic bacteria to which the above theory may hold good, *e. g.*, the tubercle bacilli, which require a temperature of 37° C. or more for their multiplication.

The reaction of the body fluids, according to the experiments of Behring, seems to have a decided influence in producing the decided immunity of the white rat to anthrax. The blood of these animals is decidedly alkaline, to which is attributed the remarkable resistance of these animals to virulent cultures of the bacillus of anthrax. When so fed, however, as to render their blood acid they readily yield to the disease. It has also been shown by Behring, Nuttall, and others that recently-drawn blood of naturally immune animals has a decided germicidal effect on certain pathogenic bacteria, while upon others it has no such effect. Buchner has demonstrated that this power belongs to the fluid part of the blood and not to the cellular elements. This power is said to be destroyed by heat, and lost when the blood has been kept for a considerable length of time, but is not affected by cold. It is also claimed that this power of the blood is very decided in its action, but limited to a given number of bacteria, and that when this number is exceeded rapid development of the germ takes place in the blood-serum, but becomes very much attenuated.

Hankins has isolated from the spleen and blood of rats a substance which has decided germicidal properties, and which he terms *globulin*. While keeping in view the alkaline theory, he attributes the power of rats' blood to destroy the anthrax bacillus largely to this globulin.

Kitasato and others have published interesting results of their experiments, in which they made a bouillon from the thymus gland of a calf. It was found that the tetanus bacillus when cultivated in this fluid did not form spores and had but little virulence. Small animals inoculated with small quantities of these cultures were rendered immune. The blood-serum of such animals was also found to be capable of producing immunity in other animals. The same was also found to be true with a few other pathogenic germs. This experiment led to the present antitoxin treatment for diphtheria. To this gland may be attributed the certain degree of immunity enjoyed by certain young.

It is worthy of note that by far the majority of pathogenic bacteria are only capable of producing disease by the products, toxalbumins, of their multiplication within the system, or by the absorption of these products through the tissues, as in diphtheria and tetanus, and that the immunitive substance in the blood or tissue must have the power of neutralizing these toxalbumins. In support of this theory Arloing has been able to produce symptomatic anthrax in naturally immune animals by adding certain chemicals to the cultures with which they were inoculated; thus seeming to indicate that the immunitive substance was destroyed by the chemical.

The subject of *acquired immunity* deserves our special attention, since the whole army of bacteriologists seemed to be centred upon that one point for distinction, and, while as yet but little is known on the subject, marvellous revelations may be looked for in the comparatively near future.

It is a well-established fact that a single attack of such diseases as smallpox, scarlet fever, and yellow fever generally confer immunity from subsequent attacks. It is interesting to note, too, that this immunity is a duration of a lifetime. In the above-mentioned diseases the system becomes affected in general, although this is not necessary to produce perfect immunity, for we have similar results from such local affections as whooping-cough and mumps. In the first instance the immunitive substance is developed within the system, while in the second it must be absorbed from the infected area. It is also worthy of note that an attack of a certain infectious disease does not render the patient immune from other infectious diseases.

A number of theories have been advanced as to the nature of this acquired immunity, but none have been adopted as clear and definite. The severity of an attack of an infectious disease does not affect the degree of immunity produced; *e. g.*, a mild attack of smallpox or scarlet fever will render the patient as thoroughly immune as a severe attack. Indeed, a severe attack of the disease does not always render the patient absolutely immune, for cases are on record in which certain individuals have been known to suffer a second attack.

For a time it was the belief of Pasteur that in an attack of an infectious disease the pathogenic bacteria in its multiplication in the body of a susceptible animal exhausted some substance necessary for its growth, and that this substance was never reproduced. In support of this theory an artificial culture media may be compared to the body. When bacteria have grown in a given quantity of culture media for a certain time, it is observed that further development ceases, which may be due to one or both of two factors, viz., exhaustion of nutrition and excessive amount of toxalbumins.

We are hardly justified in supposing that the nutrition of the body has been used up by the bacteria, and would, therefore, have to conclude that the toxalbumins present in the system prevent any further development of the germ. At the same time it is hardly reasonable to suppose that these substances would not be excreted from the body in course of time. It would be just as unreasonable to suppose that a normal substance in the body which had been exhausted by the bacteria would not again be reproduced.

It will be seen, then, that if immunity is due to either of the above theories alone, it could only be temporary. It has been argued by some, especially Metschnikoff, that immunity is largely, if not entirely, due to the action of the white blood-corpuscles which Metschnikoff terms phagocytes. When the system becomes invaded with bacteria it is known that many of the germs are picked up and destroyed by these corpuscles. It is really a battle between the leucocytes and the bacteria, in which many on each side are slain. If examined under the microscope leucocytes will be seen to contain various numbers of bacteria in their interior. In some it would appear that the leucocytes were capable of digesting the bacteria and assimilating them as nutrition, while in others the opposite suggests itself, in view of which the question naturally arises: Do the bacteria flock to the leucocytes because they find in them a suitable culture media, or do the leucocytes make the attack?

Those who support the theory of phagocytosis attribute immunity to the power of the leucocytes to attack and destroy the germ. There is no doubt that they have some such power, but I do not believe that we can attribute immunity to this one factor alone.

It is well known that the system may acquire a certain tolerance to certain drugs, such as opium, tobacco, and arsenic. When the use of these is continued for some time, doses sufficiently large to destroy the life of an ordinary individual may be taken with impunity. If the system will acquire a tolerance to the action of such drugs, it is reasonable to suppose that it may also acquire a tolerance to the action of bacteria or their toxalbumins.

Until more is known on the subject we shall have to be content with the theory that immunity is due to three factors, viz.: the action of leucocytes upon bacteria when introduced into the body; acquired tolerance of the cellular elements of the body, and the formation in the system of antitoxins which oppose further development of the pathogenic micro-organism.

